# Flumazenil Reversal of Remimazolam Sedation During Posterior Spinal Fusion in Two Adolescents

**DOI:** 10.14740/jmc5221

**Published:** 2025-12-24

**Authors:** Nikole Lee, Kelly Moon, Joshua C. Uffman, Joseph D. Tobias

**Affiliations:** a Department of Anesthesiology and Pain Medicine, Nationwide Children’s Hospital, Columbus, OH, USA; b Department of Anesthesiology and Pain Medicine, The Ohio State University College of Medicine, Columbus, OH, USA

**Keywords:** Remimazolam, Flumazenil, Benzodiazepine, Neurophysiologic monitoring, Wake-up test

## Abstract

Remimazolam is a novel, ester-metabolized benzodiazepine, which received approval by the United States Food and Drug Administration (FDA) for procedural sedation in adults in 2020. Since then, its clinical uses have expanded to intraoperative use both as the primary agent or as an adjunct to general anesthesia. Although its novel route of metabolism through tissue esterases generally results in a rapid resolution of its effects when the infusion is discontinued; in certain clinical scenarios, reversal of its clinical effects may be achieved with flumazenil. We present two clinical cases outlining the use of flumazenil to reverse the effects of remimazolam, which was used as an adjunct to total intravenous anesthesia during posterior spinal fusion (PSF) in two adolescent patients. In our first case, to facilitate an intraoperative wake-up test, the clinical effects of remimazolam were reversed with flumazenil. In the second case, flumazenil reversed the residual effects of remimazolam to speed awakening and tracheal extubation at the completion of the surgical procedure. The clinical uses of remimazolam are reviewed, experience with its use as an adjunct during PSF is discussed, and the clinical role of reversal with flumazenil is presented.

## Introduction

Remimazolam is an ultra-short acting benzodiazepine that, similar to midazolam, provides anxiolysis, amnesia, and sedation through agonism at the γ-aminobutyric acid (GABA)_A_ receptors [1]. Its novel metabolic pathway, through tissue esterases, results in a shorter half-life and a limited context-sensitive half-time (CSHT) [2-5]. As a benzodiazepine, it shares the advantage that its clinical effects can be reversed by the competitive antagonist, flumazenil, as needed to speed awakening time [6]. Remimazolam was first approved in China in 2019 for sedation during upper gastrointestinal (GI) endoscopy and was subsequently approved in 2020 by the United States Food and Drug Administration (FDA) for procedural sedation in adults [2].

In addition to its use for procedural sedation, remimazolam has been used as a primary agent or as an adjunct to both volatile and intravenous anesthetic agents during general anesthesia [7-9]. Although remimazolam is not approved by the FDA for use in pediatric patients, there is increasing off-label experience in pediatric patients for various clinical scenarios including general anesthesia during posterior spinal fusion (PSF) with neurophysiologic monitoring [10, 11]. We describe two case studies using remimazolam as part of total intravenous anesthesia (TIVA) for PSF where flumazenil was useful for two clinical indications.

This review was conducted in compliance with the ethical standards of the responsible institution on human subjects as well as with the Helsinki Declaration.

## Case Reports

### Case 1

The patient was a 12-year-old, 40.3-kg adolescent with early onset idiopathic scoliosis and no other relevant comorbid conditions, who presented for T2-L4 instrumented PSF. Following premedication with oral midazolam, anesthesia was induced by the inhalation of sevoflurane in nitrous oxide and oxygen. Following the induction of anesthesia, a peripheral intravenous catheter was placed, and the depth of anesthesia was deepened by the administration of propofol and fentanyl. A single dose of rocuronium (0.4 mg/kg) was administered to facilitate endotracheal intubation. Following endotracheal intubation, a second peripheral intravenous cannula and a radial arterial cannula were placed. Due to the plan for neurophysiologic monitoring with somatosensory and motor evoked potentials (SSEP and MEP) during surgery, maintenance anesthesia included methadone (0.1 mg/kg) followed by a remifentanil infusion at 0.1 - 0.4 µg/kg/min and propofol, starting at 150 µg/kg/min. To decrease propofol infusion requirements, a remimazolam infusion was started at 5 µg/kg/min after the patient was positioned prone on the Jackson table. Based on depth of anesthesia monitoring using the Bispectral Index (BIS), maintained at 50 - 60, the propofol infusion was decreased incrementally to 75 µg/kg/min and then to 50 µg/kg/min. Approximately, 5 - 6 h into the case, due to signaling changes on neurophysiologic monitoring, specifically a decrease in MEPs, the surgical team activated the protocol followed at our center for loss of neuromonitoring potentials. Following an increase of the mean arterial blood pressure to ≥ 70 mm Hg and removal of recently placed surgical screws, there was a return of the neurophysiologic parameters to baseline. Approximately 90 min later, there was a second episode of loss of MEPS. Despite repetition of the previous interventions, no improvement was achieved, prompting the decision to conduct a wake-up test. The propofol, remimazolam, and remifentanil infusions were paused. Eight minutes later, as the patient was not following commands, flumazenil (0.1 mg) was administered followed by a second dose (0.2 mg) 2 min later, as the first dose did not result in an adequate awakening to allow the patient to follow commands. Three minutes after administration of the second flumazenil dose, the patient demonstrated the ability to follow commands and exhibited voluntary movement of the upper, but not the lower extremities. The surgical team evaluated potential surgical causes, and the remifentanil infusion was reinitiated at 0.1 µg/kg/min in conjunction with intermittent propofol boluses to maintain amnesia. Corticosteroid therapy was started per our protocol, but given the persistent absence of MEPs, a second wake-up test was initiated 12 min later. At that time, the remifentanil and propofol infusions were discontinued, and an additional dose of flumazenil (0.1 mg) was administered to facilitate emergence. Following the second wake-up test, satisfactory motor function of all extremities was confirmed. Thereafter, anesthesia was re-established with a bolus dose of propofol and the continuous infusions of propofol, remifentanil, and remimazolam were resumed. The remainder of the procedure was uneventful. Total surgical duration was 7 h 40 min. Intraoperative fluid administration included 500 mL of colloid (5% albumin), 1,200 mL of crystalloids, and 530 mL of blood products (packed red blood cells and cell saver autologous blood). Estimated blood loss was 750 mL. At the completion of the surgery, the patient was turned supine, and her trachea was extubated when awake. She was transported to the post-anesthesia care unit (PACU) and then the pediatric intensive care unit (ICU). Her postoperative neurologic examination was normal. The postoperative course was uneventful.

### Case 2

The patient was a 13-year-old, 51-kg adolescent with early onset idiopathic scoliosis and no other relevant comorbid conditions, who presented for PSF. The patient was transported to the operating room, routine American Society of Anesthesiologists monitors were placed, and anesthesia was induced by the inhalation of sevoflurane in nitrous oxide and oxygen. Following the induction of anesthesia, a peripheral intravenous catheter was placed, and the depth of anesthesia was deepened by the administration of propofol, midazolam, and fentanyl. A single dose of rocuronium was administered to facilitate endotracheal intubation. A second peripheral intravenous cannula and a radial arterial cannula were placed. Due to the plan for neurophysiologic monitoring (SSEP and MEP) during surgery, maintenance anesthesia included methadone (0.1 mg/kg), remifentanil 0.3 µg/kg/min, and propofol, starting at 130 µg/kg/min. To decrease propofol infusion requirements, a remimazolam infusion was started at 10 µg/kg/min. Based on depth of anesthesia monitoring using the BIS, the propofol infusion was decreased incrementally to 80 µg/kg/min. When the first PSF rod was locked in place, the remimazolam infusion was decreased to 5 µg/kg/min, and then both the remimazolam and propofol infusions were discontinued when the second rod was locked in place and the final set of MEPs were obtained. For the final 50 min of the surgical procedure, anesthesia was maintained with inspired desflurane in air/oxygen, titrated to the maintain the BIS at 50 - 60. At the completion of the surgical procedure, the desflurane and remifentanil infusions were discontinued, and the patient was turned supine. In 5 min, as the patient was not fully responsive, flumazenil (0.1 mg) was administered intravenously, followed by a second dose (0.1 mg) 1 min later. Within 1 min of the second dose, the patient was fully responsive and following commands. Her tracheal was extubated, and she was transferred to the PACU and then the inpatient ward. Her postoperative course was unremarkable.

## Discussion

As a novel, ester-metabolized benzodiazepine, remimazolam has seen increased use in various clinical scenarios since its approval by the FDA for procedural sedation in adults in 2020. Although its novel route of metabolism through tissue esterases can generally be expected to result in a rapid resolution of its effects when the infusion is discontinued; in specific clinical scenarios, reversal of its sedative properties may be achieved with flumazenil. We present two clinical cases outlining the use of flumazenil to reverse the effects of remimazolam, following its use as an adjunct to TIVA during PSF in two adolescents. In our first case, flumazenil was administered to facilitate an intraoperative wake-up test when there were changes noted in neurophysiologic monitoring parameters. In our second patient, flumazenil was administered at the completion of the case to speed awakening and tracheal extubation following the surgical procedure.

Although the incidence of neurologic deficits following surgical procedures on the vertebral column has been estimated to be as high as 3.7-6.9% without neurophysiologic monitoring, the incidence can be decreased to less than 1% with appropriate monitoring [12, 13]. In their guidelines on intraoperative monitoring, the American Academy of Neurology concluded that the evidence favors the use of monitoring as a safe and efficacious tool in clinical situations where there is a significant nervous system risk. A recent meta-analysis of multimodality spinal cord monitoring (MEP and SSEP) during idiopathic scoliosis surgery that included seven studies and 2,052 patients reported that the incidence of neurological deficits was 0.93% [13]. All 19 patients with a neurological deficit had a change in SSEP and MEP. No patient without neuromonitoring change had a deficit. The overall sensitivity for predicting neurological deficit when both modalities were utilized was 83%, with a specificity of 94%, a positive predictive value of 12%, and a negative predictive value of 99.8%. One of the major limitations is that the window of opportunity - from the time when changes in monitoring parameters are noted until permanent damage occurs - may be less than 10 min. Additionally, as was the case in our patients, when these techniques are used, specific modifications of the anesthetic technique are needed, generally using a TIVA with a propofol-based technique.

The wake-up test, historically used for the detection of intraoperative spinal cord injury, has become less routine with the advent of multimodality neuromonitoring. However, it remains an important rescue tool when neuromonitoring signals are equivocal or lost. In our first case, the decision to perform a wake-up test was prompted by persistent changes in neurophysiologic monitoring parameters despite optimization of the intraoperative and surgical conditions, including assurance of adequate perfusion pressure and oxygen delivery. Although no longer performed routinely, the wake-up test continues to have a role as a confirmatory measure when neuromonitoring data cannot be reliably interpreted or when changes cannot be reversed with optimization of physiologic parameters.

The choice of anesthetic agents during spine fusion surgery with neuromonitoring is critical, as they can influence the signal quality and impact recovery during a wake-up test [14]. Although propofol-based TIVA is generally used, concerns with propofol include a prolonged CSHT with delayed awakening following longer term infusions [15]. To mitigate these concerns, there is interest in the use of remimazolam as an adjunct to TIVA with propofol to decrease propofol infusion requirements and perhaps decrease its CSHT, which may result in delayed awakening [11]. The largest study to date outlining the use of remimazolam during neurophysiologic monitoring included a retrospective review of 40 patients (11 to 35 years of age) [10]. The cohort included 11 patients who received TIVA with propofol and remimazolam as an adjunct during PSF. With the addition of remimazolam, the propofol infusion requirements to maintain the BIS at 50 - 60 decreased from 150 - 200 µg/kg/min to 70 - 100 µg/kg/min. Moreover, unlike volatile anesthetic agents, remimazolam does not impact MEP or SSEP responses, allowing reliable monitoring and can be successfully incorporated into a TIVA regimen for neurophysiologic monitoring during PSF.

A unique feature of remimazolam compared to other commonly used intravenous agents is the availability of a specific antagonist, flumazenil. In our two cases, reversal of the sedative effects of remimazolam with flumazenil allowed for a prompt awakening to perform the required wake-up test, as well as speeding emergence at the completion of the surgical procedure in our second patient, thereby allowing tracheal extubation and a postoperative neurologic examination. This capability may provide an additional margin of safety in procedures where rapid neurologic assessment is needed. Previous reports in adults have also described the successful use of flumazenil to reverse remimazolam sedation in various clinical scenarios [15-21].

While flumazenil is generally well tolerated, its use is not without risk. A key concern is the potential for re-sedation, as the elimination half-life of flumazenil is shorter than that of most benzodiazepines, including remimazolam [22-24]. Although both drugs are reported to have comparable terminal half-lives according to the FDA package insert, the clinical risk of recurrent sedation remains. Flumazenil may also lower the seizure threshold in susceptible individuals, particularly those with chronic benzodiazepine exposure or a history of seizure disorders. In a meta-analysis by Penninga et al including 994 patients with suspected benzodiazepine overdose, the most frequent serious adverse events associated with flumazenil were supraventricular arrhythmias and convulsions [22]. However, it remains unclear whether these events represent direct pharmacological effects of the antagonist or are related to the abrupt reversal of benzodiazepine activity. The same study also found agitation to be the most common adverse event, raising additional concerns about its potential to precipitate agitation and emergence delirium. More recently, Komatsu et al conducted a large retrospective study of 12,033 patients who underwent surgery with remimazolam reversed by flumazenil, comparing outcomes with a propofol cohort [23]. Notably, no significant differences in seizure incidence were observed between the two groups, suggesting that the overall risk of seizures with flumazenil may be lower in the perioperative setting. Although no adverse effects were observed in our patients and their subsequent postoperative course and recovery was uneventful, careful monitoring after reversal remains essential as does consideration of the adverse effect profile of flumazenil.

### Learning points

In summary, this case highlights several important considerations in the anesthetic management of PSF with neuromonitoring. Our practice has shifted toward using remimazolam as an adjunct to decrease propofol requirements for general anesthesia and thereby limit its associated CSHT. In such settings, flumazenil may be a useful adjunct to reverse the sedative effects of remimazolam and facilitate awakening during a wake-up test, mandated by changes in neurophysiologic monitoring, or to decrease the time to rapid tracheal extubation at the completion of the procedure. In these scenarios, incremental doses of 0.01 - 0.02 mg/kg to a maximum dose single bolus dose of 0.1 - 0.2 mg can be administered, up to a total dose of 0.5 mg to achieve the desired decrease in the depth of anesthesia/sedation. Further studies are warranted to better define the role of remimazolam and flumazenil in pediatric-aged patients, particularly during procedures requiring neurophysiologic monitoring or rapid emergence.

## Data Availability

Any inquiries regarding supporting data availability of this study should be directed to the corresponding author.
